# Ultrasound for Measuring the Cross-Sectional Area of Biceps Brachii Muscle in Sarcopenia

**DOI:** 10.7150/ijms.49637

**Published:** 2020-10-18

**Authors:** Shumin Li, Hanyu Li, Ying Hu, Shaoming Zhu, Zherong Xu, Qin Zhang, Yunmei Yang, Zhaodi Wang, Jia Xu

**Affiliations:** 1 Department of Geriatrics, the First Affiliated Hospital, School of Medicine, Zhejiang University, Hangzhou, 310003, China; 2 Department of Ultrasound, The First Affiliated Hospital, School of Medicine, Zhejiang University, Hangzhou, 310003, China; 3 Zhejiang Provincial Key Laboratory for Diagnosis and Treatment of Aging and Physic-chemical Injury Diseases, the First Affiliated Hospital, School of Medicine, Zhejiang University, Hangzhou, 310003, China; 4 Department of Emergency Medicine, The First Affiliated Hospital, School of Medicine, Zhejiang University, Hangzhou, 310003, China

**Keywords:** sarcopenia, ultrasound, biceps brachii, muscle mass

## Abstract

**Background:** Ultrasound is emerging as an effective method for measuring muscle mass in elderly people. It has been applied in numerous studies to obtain measurement of lower limbs. The study aims to explore the relationship between sarcopenia and ultrasound measurements of biceps brachii.

**Methods:** Participants (n=179) aged over 60 years were enrolled from the first affiliated hospital of Zhejiang University. The muscle thickness (MT), cross-sectional area (CSA) and fat thickness (FT) of these participants were recorded. Spearman test and partial correlation test was used to determine the correlation between indicators. Mann-Whitney U test was performed to compare ultrasonic parameters between sarcopenia group and non-sarcopenia group. The binary logistic regression analysis was employed to detect the potential indicators and prediction equation of sarcopenia. Receiver operating characteristic (ROC) curve analysis was performed for the accuracy of equation.

**Results:** The prevalence of sarcopenia were 16.3% and 10.8% respectively in men and women. CSA was significantly lower in sarcopenia group than non-sarcopenia group in women (*P*<0.05). CSA was positively correlated with skeletal muscle mass index (SMI) and grip strength (men: r=0.460, 0.433; women: r=0.267, 0.392). After controlling of age and BMI, these correlations disappeared. Binary logistic regression analysis showed that age (OR=1.149, 95%CI: 1.060-1.246; *P*=0.001) and CSA (OR=0.465, 95%CI: 0.225-0.963; *P*=0.039) was significant indicators associated with sarcopenia. Area Under Curve was 0.822 (95%CI: 0.725-0.919, P<0.001) for the prediction equation composed of age, gender and CSA for sarcopenia.

**Conclusion:** CSA of the biceps brachii measured with ultrasound is an important indicator associated with sarcopenia.

## Introduction

Sarcopenia, first proposed by Irwin Rosenberg in 1989, is characterized by a progressive loss of skeletal muscle mass and is associated with aging^1^. It decreases muscle strength and function. It was until 2016 when the Centers for Disease Control and Prevention approved the international classification of sarcopenia as an independent disease^2^. Sarcopenia severely deteriorates the health of elderly people and increases the risks of falls, fractures, disability, and mortality, especially in those with chronic diseases^3^. The diagnostic criteria for sarcopenia are not yet uniform. The diagnose index of sarcopenia includes the skeletal muscle mass, muscle strength (usually reflected by grip strength) and physical performance (usually reflected by gait speed)^4^-^7^. Because skeletal muscle mass is considered as one of the important indicators for sarcopenia, its measurement methods have received widespread attention. Dual-energy X-ray absorptiometry (DXA) and bio-electrical impedance analysis (BIA) are widely used to assess skeletal muscle mass. Computed tomography (CT) and magnetic resonance imaging (MRI) have been recommended as the gold standard for assessing muscle mass because they can clearly show the tissue structure. Ultrasound, being convenience and pocket-friendly, is increasingly being valued in the evaluation of skeletal muscle mass.

Although the literature and guideline suggest that the gold standard for muscle mass testing is CT or MRI^8^, ^9^, these two measurements have some limitations. They are time-consuming, expensive and require specialized equipment. The CT can even cause radiation to people. Due to these limitations, the use of CT and MRI evaluating muscle mass in the elderly has not yet peaked. Although the cut-off points of CT for sarcopenia had been reported, the practical application is still limited^10^. Although DXA has been widely accepted as the measurement of skeletal muscle mass, it has some limitations, such as radiation, cannot operate at bedside and deficiency in primary hospital. The BIA is a simple and convenient technology that is easily accepted and can operate at the bedside. Its reliability is however affected by various factors, such as an electrode, operator, environment and different machines^11^. In addition, the assessment results of DXA and BIA are different which causes nonuniform standard for sarcopenia to diagnose^12^. Therefore, there is an urgent need for a standard and uniform measurement of sarcopenia diagnose.

Ultrasound is widely used to diagnose and follow-up in the clinic. It can distinguish muscle tissue from subcutaneous fat and show the thickness and cross-sectional area of muscle. Ultrasound is a convenient, reliable and non-radiative technique, and can be performed at the bedside for those who cannot cooperate with DXA, CT or MRI. Compared with the above methods, the application of ultrasound is more common, and many people are more willing to accept the ultrasound examination, which has a great prospect in the application for sarcopenia. It has been pointed out as an effective method to assess muscle mass in the elderly^13^. Good consistency between the thickness of the thigh muscle measured by ultrasound and the muscle mass by DXA has been shown^14^.

The commonly used indices for assessing muscle mass by ultrasound include muscle thickness (or diameter), muscle cross-sectional area, muscle fascicle length, pinnation angle and muscle echo^15^. Most of the current studies on the evaluation of sarcopenia by ultrasound have focused on lower limb ultrasound, especially the quadriceps femoris and gastrocnemius muscles^15^, ^16^. Few studies have focused on the importance of assessing upper limbs using ultrasound. For a better understanding of the biceps muscle thickness (MT), cross-sectional area (CSA) and fat thickness (FT) measured by ultrasound related to sarcopenia, this study analyzed the relationship among FT, MT and CSA of biceps and muscle mass, grip strength and sarcopenia. The aim of the study is to provide a theoretical basis for the application of ultrasound in sarcopenia in the future.

## Methods

### Participants

All participants were from the health management center of the first affiliated hospital of Zhejiang University medical college. History and clinical examination of all participants were assessed. All the participants were more than 60 years old and willing to accept the examinations of DXA and ultrasound. The elderly with restricted activities such as limb hemiplegia or limb fracture within three months, muscle-related diseases such as myositis, progressive muscular dystrophy, and myasthenia gravis; dementias and those who could not cooperate with the test were excluded. A total of 179 participants were sampled for the study. The study was approved by the ethics committee and informed consent obtained from all the participants.

### Ultrasound measurements

The biceps FT, MT, and CSA of all participants were measured by the same examiner^17^. The participants were in the supine position with their limbs extended and relaxed. The examiner held the ultrasound probe (2-10 MHz, Aixplorer; Aix-en-Provence, France) vertically against the skin surface to measure accurately to 0.01 cm the maximum cross-sectional area of the biceps brachii muscle in the dominant hand. It was used as a marker to detect its axial view of Fat thickness and muscle thickness. The measurement was repeated after a 10-minute rest. The average measurement was calculated and used in the analysis.

### Muscle mass

The muscle mass of participants was assessed by DXA (DISCOVERY-W, Hologic, Bedford, MA, USA). The participants were requested to take off their clothes and the measurements were taken without metal to eliminate artifacts that could be caused by the clothes. Body composition was assessed by scanning the body. Skeletal muscle mass index (SMI) was analyzed and recorded using height-adjusted muscle mass (limb muscle mass / height²) accurate to 0.1 kg/m squared.

### Grip strength

JAMAR manual dynamometer (Asimow Engineering, Los Angeles, CA, USA) was used to measure grip strength as per the procedures by Roberts et al^18^. The participant sat comfortably on a standard chair (with backrest and flat armrests) with their forearms resting on the armrests. The participant was requested to squeeze the JAMAR until the pointer stopped rising. Measurements were taken alternately with each hand, three times on each side. The highest result was recorded, accurate to 0.1kg.

### Gait speed

A 4-meter walking test was performed to measure gait speed 19. Participants were asked to stand still behind a marked line. They started walking at their normal pace and stopped near the finish line. The maximum value of the two measurements was taken for evaluation, and the recorded value was accurate to 0.1m/s.

### Group Definition

The cut-off point recommended by the Asian sarcopenia working group was used as the diagnostic criteria 4. The SMI less than 7.0kg / m² in men and less than 5.4kg / m² in women belonged to the low muscle mass. The grip strength below 26kg and 18kg in men and women respectively belonged to the low grip strength. Low muscle mass, along with low grip strength or low gait speed (< 0.8m/s, both men and women), belonged to the sarcopenia group, and the rest belonged to the non-sarcopenia group.

### Statistical analyses

SPSS 24.0 statistical software was employed to analyze the data. The Shapiro-Wilk test was performed to test the normality. Since most of the data did not conform to the normal distribution, the Mann-Whitney U test was performed to compare two groups. The experimental data were shown as median and quartile (M(P25, P75)). Chi-square test is used for categorical data. Spearman test was used for correlation analysis. Partial correlation test was used for analysis after controlling of age and BMI. Binary logistic regression analysis was used to detect the potential indicators and prediction equation for sarcopenia. Based on the result of binary logistic regression and formula of logistic model: logit(*P*)= In [*P*/(1-*P*)] = β_0_ + β_1_X_1_ +… + βnXn (the value of β comes from logistic regression, X is the independent variable, n is the number of independent variables), we can get the value of probability for dependent variable: *P* = e^logit(*P*)^ /1+e^logit(*P*)^. The accuracy of equation is assessed by receiver operating characteristic (ROC) curve analysis. The statistical value of p < 0.05 was used to test significance.

## Results

A total 179 participants were recruited, 0 was excluded. There were 8 participants in men and 14 participants in women who were diagnosed as sarcopenia. The prevalence of sarcopenia was 16.3% and 10.8% respectively in men and women. Men were significantly higher than women in terms of height, weight, BMI, muscle mass, grip strength, MT, and CSA. While FT was significantly lower in men than in women. There was no significant difference in age and gait speed between two groups (Table 1). The baseline information between sarcopenia group and non-sarcopenia group is shown at Supplementary Table 1.

For women, the FT and CSA were significantly higher in the non-sarcopenia group than those in sarcopenia group. No statistically significant differences in ultrasound parameters were found between two groups in men (Table 2).

A negative correlation between age and grip strength, gait speed, MT and CSA was found in both men and women. A positive correlation was observed between CSA and SMI or grip strength. (Table 3) After controlling of age and BMI, correlations between SMI, grip strength, gait speed, and ultrasound parameters are not significant.

Age, gender, BMI, FT, MT, and CSA were included in the binary logistic regression analysis for sarcopenia. It showed that age (OR=1.149, 95%CI: 1.060-1.246; *P*=0.001) and CSA (OR=0.465, 95%CI: 0.225-0.963; *P*=0.039) were significant indicators associated with sarcopenia (Table 4).

**Figure 1:**
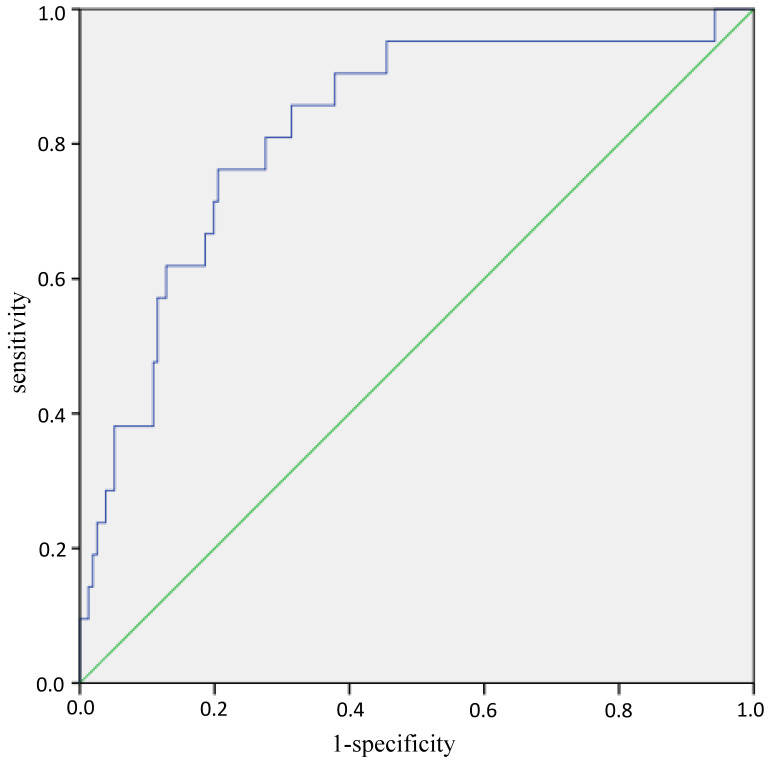
ROC curves of the equation in predicting sarcopenia. Note: The blue line is the tracing of ROC analysis of equation in predicting sarcopenia, the green line is the baseline. Abbreviations: ROC, receiver operating characteristic.

Age, gender and CSA were included into the binary logistic regression analysis again to establish the prediction equation for sarcopenia: logit(*P*) = -7.542 + 0.125*age - 1.584*gender (man=0, woman=1) - 0.449*CSA, *P* = e^logit(*P*)^ /1+e^logit(*P*)^. ROC curves analyses showed that AUC (Area Under Curve) was 0.822 (95%CI: 0.725-0.919, *P*<0.001) (Figure 1). While the AUC was 0.802 (95%CI: 0.688-0.916, P<0.001) when the equation consists of BMI, FT, MT and CSA (Supplementary Figure 1).

When grip strength of cut-off point for sarcopenia was 28 kg in men according to the latest recommendation by the Asian sarcopenia working group 20, the differences between sarcopenia group and non-sarcopenia group were listed at supplementary table 2. The age (OR=1.131, 95%CI: 1.048-1.220; *P*=0.001), gender (OR=0.124, 95%CI: 0.023-0.682; *P*=0.016) and CSA (OR=0.422, 95%CI: 0.207-0.861; *P*=0.018) were significant indicators associated with sarcopenia (Supplementary Table 3). AUC was 0.842 (95%CI: 0.752-0.933, P<0.001) of the prediction equation consisting of age, gender and CSA (Supplementary Figure 2).

## Discussion

The prevalence of sarcopenia was 1-29% for the elderly who live in the community and 14-33% in residents requiring long-term care according to the definition of European Working Group on Sarcopenia in Older People.^21^ And the prevalence was reported to be 2.5-28.0% in men and 2.3-11.7% in women for the community‐dwelling Japanese elderly.^22^ In the study, the prevalence of sarcopenia was 16.3% and 10.8% respectively in men and women, which is accord with the previous reports. The high prevalence of sarcopenia in the elderly reminds us to pay more attention.

At present, the use of ultrasound to evaluate the muscle mass of the elderly has become a research hotspot. It's reported a good homogeneity of diameter and cross-sectional area of the rectus femoris measured by ultrasound and CT^23^. The similar results were confirmed by the contrast between ultrasound and MRI^24^. From these previous findings, the ultrasound could be proposed to be an effective measurement for assessing muscle mass. Here, we mainly assess the value of ultrasound measurement of the biceps brachii in sarcopenia.

Considering gender indicators, the study found the muscle mass and grip strength of men to be significantly greater than that of women, while the gait speed was not significantly different between the two groups. The MT and CSA of biceps brachii in man were significantly higher than those in women, while FT was significantly lower than those in women. This to some extent reflects the differences in muscle and adipose tissue distribution between men and women, and men seem to have more muscle tissue and less adipose tissue than women as previously stated^25^.

Age is an independent risk factor for sarcopenia. The older the person gets, the greater the risk of sarcopenia. It has been reported that after 50 years of age, muscle mass decreases at a rate of about 1% to 2% per year, while muscle strength decreases at a rate of about 1.5% per year, and gradually accelerates to 3% per year after 60^3^. A prospective study in Asia also found that muscle mass and gait speed progressively decrease as humans get older^26^. In this study, binary logistic regression analysis showed that age was a significant factor associated with sarcopenia. Correlation analysis also revealed a negative correlation between age and grip strength or gait speed. Age and SMI also showed a significant negative correlation in elderly men, which is in line with the results of Japanese studies^27^. Moreover, the biceps brachii MT and CSA were negatively correlated with age.

Previous studies have shown a good correlation between ultrasound assessment of muscle and skeletal muscle mass measured by DXA^28^-^30^. Ismail et al^31^ found a positive correlation between the muscle thickness measured by ultrasound and skeletal muscle mass measured by DXA. In addition to muscle mass, a strong correlation was observed between ultrasound parameters and muscle strength. 32. The muscle echo strength had a significant positive correlation with grip strength 31. Loss of muscle mass in specific parts of thighs assessed by ultrasound may be related to a decreased curve walking performance^33^. The muscle thickness of the forearm of the upper limb was also significantly correlated to the grip strength^34^.In present study, positive correlations between CSA of the biceps brachii and SMI or grip strength were observed in both genders. But after controlling of age and BMI, the correlations between SMI and ultrasound parameters are not significant. These results indicated that the correlations between biceps ultrasound parameters and SMI or grip strength are associated with age and BMI.

The study by Kuyumcu et al^35^ found the sarcopenia group defined by BIA and grip strength have significantly lower ultrasonic parameters of muscle thickness and fascicle length of gastrocnemius muscles than the non-sarcopenia group. Research also found that the thickness of the gastrocnemius muscle could effectively assess and predict low muscle mass^36^. Seymour et al^37^ reported a positive correlation between the cross-sectional area of the rectus femoris measured by ultrasound and that by CT, as well as the ultrasound parameters and the skeletal muscle mass measured by BIA. A strong positive correlation between the ulnar muscle thickness of the forearm measured by ultrasound and the skeletal muscle mass measured by DXA has been reported. 34, 38 All these data reflect the importance of ultrasound in sarcopenia. Our study found that biceps brachii CSA was significantly higher in the non-sarcopenia group than those in sarcopenia group. CSA as well as age was significant indicator for sarcopenia. Moreover, the equation of age, gender and CSA was a feasible method to predict the sarcopenia, which wasn't reported before.

### Limitation

Although the study established the significance of biceps brachii CSA in the sarcopenia, the low number of participants and restricted region might have caused a certain degree of result bias. A larger data including multiple geographic regions and a range of health states is needed to identify clinically relevant thresholds for sarcopenia. The study just focuses on the biceps brachii, other sections of body isn't detected, which needs more studies to determine. The study mainly focuses on the value of ultrasound, the diet and exercise hadn't been investigated and the relationship between diseases or biochemical data and sarcopenia hadn't been explored.

## Supplementary Material

Supplementary figures and tables.

## Figures and Tables

**Table 1:** Differences between gender

	Men(n=49)	women(n=130)	P
General information			
age(year)	70(66, 80)	69(64, 77)	0.180
Height(cm)	169(166, 174)	156(152, 160)	***<0.001***
Weight(kg)	70.0(61.5, 79.0)	57.8(51.7, 62.0)	***<0.001***
BMI (kg/m²)	24.2(22.4, 27.1)	23.4(21.7, 25.4)	**0.045**
Sarcopenia assessment			
SMI (kg/m²)	6.3(5.7, 7.5)	5.3(4.5, 6.3)	***<0.001***
Grip strength(kg)	35.9(32.5, 40.3)	22.9(20.0, 26.0)	***<0.001***
Gait speed(m/s)	1.2(1.0, 1.4)	1.2(1.0, 1.3)	0.052
Ultrasound assessment			
FT (cm)	0.23(0.16, 0.31)	0.33(0.25, 0.61)	***<0.001***
MT (cm)	2.14(2.03, 2.48)	1.69(1.47, 1.90)	***<0.001***
CSA (cm^2^)	8.88(7.03, 10.36)	5.46(4.50, 6.05)	***<0.001***

Notes: Mann-whitey U test was used for comparison between two groups. The data were shown as median and quartile (M(P25,P75)). Bold means P <0.05, bold + italics means P <0.01. Abbreviations: BMI, body mass index; SMI, skeletal muscle mass index; FT, fat thickness; MT, muscle thickness; CSA, cross-sectional area.

**Table 2:** differences between sarcopenia group and non-sarcopenia group

	Non-sarcopenia group	sarcopenia group	
male	n=41	n=8	P
age(year)	69(65, 77)	79(74, 83)	**0.013**
BMI (kg/m²)	25.1(23.1, 27.4)	21.8(19.5, 23.7)	***0.003***
SMI (kg/m²)	6.5(5.8, 7.7)	5.5(4.8, 5.8)	***0.002***
Grip strength(kg)	37.1(33.8, 42.0)	26.4(17.0, 32.5)	***<0.001***
Gait speed(m/s)	1.3(1.1, 1.4)	0.7(0.6, 1.1)	***0.007***
FT (cm)	0.26(0.18, 0.33)	0.20(0.14, 0.25)	0.386
MT (cm)	2.20(2.03, 2.63)	2.08(1.97, 2.11)	0.158
CSA (cm^2^)	9.24(7.37, 10.67)	7.06(6.40, 9.00)	0.075
female	n=116	n=14	P
age(year)	68(64, 76)	80(74, 82)	***0.001***
BMI (kg/m²)	23.4(22.0, 25.5)	22.5(20.1, 24.6)	0.150
SMI (kg/m²)	5.4(4.7, 6.5)	4.5(4.1, 5.0)	***0.001***
Grip strength(kg)	23.3(20.7, 26.3)	16.4(12.6, 18.2)	***<0.001***
Gait speed(m/s)	1.2(1.1, 1.3)	0.8(0.6, 0.9)	***<0.001***
FT (cm)	0.35(0.26, 0.42)	0.23(0.19, 0.31)	***0.001***
MT (cm)	1.71(1.47, 1.92)	1.61(1.33, 1.74)	0.080
CSA (cm^2^)	5.50(4.60, 6.12)	4.40(3.34, 5.49)	***0.003***

Notes: Mann-whitey U test was used for comparison between two groups. The data were shown as median and quartile (M(P25,P75)). Bold means P <0.05, bold + italics means P <0.01. Abbreviations: BMI, body mass index; FT, fat thickness; MT, muscle thickness; CSA, cross-sectional area.

**Table 3:** The correlation between indicators

correlation	age	BMI	SMI	Grip strength	Gait speed	FT	MT	CSA
male								
Age	1.000	-0.121	***-0.419***	***-0.566***	**-0.357**	0.029	**-0.307**	**-0.319**
BMI		1.000	***0.479***	**0.337**	0.121	***0.628***	***0.431***	***0.501***
SMI			1.000	**0.347**	0.162	0.268	**0.359**	***0.460***
Grip strength				1.000	0.128	0.144	***0.399***	***0.433***
Gait speed					1.000	0.093	0.122	0.097
FT						1.000	0.226	0.107
MT							1.000	***0.841***
CSA								1.000
female								
Age	1.000	-0.028	*-0.030*	***-0.384***	**-0.505**	-0.106	***-0.235***	***-0.318***
BMI		1.000	***0.327***	0.013	0.060	***0.491***	***0.245***	***0.246***
SMI			1.000	0.109	0.052	***0.226***	0.121	***0.267***
Grip strength				1.000	***0.459***	0.145	***0.317***	***0.392***
Gait speed					1.000	0.080	0.159	**0.214**
FT						1.000	***0.463***	***0.249***
MT							1.000	***0.749***
CSA								1.000

Notes: Spearman test was used for correlation analysis. Bold means P <0.05, bold + italics means P <0.01. Abbreviations: BMI, body mass index; SMI, skeletal muscle mass index; FT, fat thickness; MT, muscle thickness; CSA, cross-sectional area.

**Table 4:** Binary logistic regression analysis for sarcopenia

variable	B	Wald	P	OR	95%CI
Gender (women)	-0.908	0.991	0.320	0.403	0.067-2.411
**Age(year)**	**0.139**	**11.472**	**0.001**	**1.149**	**1.060-1.246**
BMI	-0.152	1.602	0.206	0.859	0.678-1.087
FT(mm)	-0.588	2.848	0.091	0.556	0.281-1.099
MT(mm)	0.268	2.947	0.086	1.307	0.963-1.775
**CSA(cm2)**	**-0.765**	**4.245**	**0.039**	**0.465**	**0.225-0.963**

Notes: age, sex, BMI, MT, FT, and CSA were included in the binary logistic regression analysis of sarcopenia. Abbreviations: BMI, body mass index; FT, fat thickness; mm, millimeter; MT, muscle thickness; CSA, cross-sectional area; cm, centimeter.

## Abbreviations

DXA: Dual-energy X-ray absorptiometry
BIA: bio-electrical impedance analysis
CT: computed tomography
MRI: magnetic resonance imaging
MT: muscle thickness
CSA: cross-sectional area
FT: fat thickness
ROC: receiver operating characteristic
AUC: Area Under Curve
