# Supplementary material for: Ultrasound for Measuring the Cross-Sectional Area of Biceps Brachii Muscle in Sarcopenia
Source: Int J Med Sci. 2020 Oct 18;17(18):2947–53. doi: 10.7150/ijms.49637 (PMC7646111; doi:10.7150/ijms.49637)
Supplement: Supplementary file 1 — Supplementary figures and tables. [file ijmsv17p2947s1.pdf]

Supplementary material

Table 1. Baseline information between sarcopenia group and non-sarcopenia group

	Non-sarcopenia group (n=157)	sarcopenia group (n=22)	*P*
**General information**			
Gender (men) (n, %)	41, 26.1%	8, 36.4%	0.313
Age (year)	68 (64, 77)	79 (74, 82)	***<0.001***
BMI (kg/m^2)	23.8 (22.3, 25.8)	22.1 (20.1, 24.3)	***0.005***
**Chronic diseases**			
Diabetes (n, %)	26, 16.6%	4, 18.2%	1.000
Hypertension (n, %)	80, 51%	12, 54.5%	0.752
coronary heart disease (n, %)	34, 21,7%	8, 36.4%	0.127
Chronic renal dysfunction (n, %)	3, 1.9%	4, 18.2%	***0.005***
osteoporosis (n, %)	68, 43,3%	8, 36.4%	0.537
**Biochemical data**			
Hemoglobin (g/L)	140 (134, 149)	138 (132, 143)	0.343
Albumin (g/L)	47.3 (45.8, 49.0)	45.8 (44.0, 46.9)	***0.001***
Creatinine (umol/L)	69 (61, 81)	77.5(59, 89)	0.166
Fasting glucose (mmol/L)	5.34 (4.89, 5.82)	5.39 (4.90, 6.28)	0.433

Notes: Mann-whitey U test was used for comparison between two groups. The data were shown as median and quartile (M(P25,P75)). Chi-square test is used for categorical data. Bold means P <0.05, bold + italics means P <0.01.

Abbreviations: BMI, body mass index.

Table 2. differences between sarcopenia group and non-sarcopenia group when grip strength of cut-off point for sarcopenia was 28 kg in male.

	Non-sarcopenia group	sarcopenia group	
male	n=39	n=10	P
Age	68(65, 77)	79(73, 83)	***0.008***
BMI	25.1(23.1, 27.5)	21.9(20.7, 24.4)	***0.009***
SMI	6.7(5.8, 7.8)	5.7(5.1, 6.1)	***0.007***
Grip strength	37.6(34.3, 43.1)	27.0(19.7, 32.4)	***<0.001***
Gait speed	1.3(1.1, 1.5)	0.9(0.6, 1.2)	***0.006***
FT	0.26(0.18, 0.34)	0.20(0.15, 0.27)	0.449
MT	2.21(2.05, 2.65)	2.06(1.88, 2.11)	**0.017**
CSA	9.32(7.61, 10.75)	7.03(6.12, 8.09)	***0.005***

Notes: Mann-whitey U test was used for comparison between two groups. The data were shown as median and quartile (M(P25,P75)). Bold means P <0.05, bold + italics means P <0.01.

Abbreviations: BMI, body mass index; FT, fat thickness; MT, muscle thickness; CSA, cross-sectional area.

Table 3. Binary logistic regression analysis for sarcopenia when grip strength of cut-off point for sarcopenia was 28 kg in male.

variable	B	Wald	P	OR	95%CI
**Gender （women）**	**-2.088**	**5.761**	**0.016**	**0.124**	**0.023-0.682**
**Age(year)**	**0.123**	**10.114**	**0.001**	**1.131**	**1.048-1.220**
BMI	-0.092	0.642	0.423	0.913	0.730-1.142
FT(mm)	-0.494	2.140	0.144	0.610	0.315-1.183
MT(mm)	0.176	1.368	0.242	1.193	0.888-1.603
**CSA(cm2)**	**-0.863**	**5.627**	**0.018**	**0.422**	**0.207-0.861**

Notes: age, sex, BMI, MT, FT, and CSA were included in the binary logistic regression analysis of sarcopenia.

Abbreviations: BMI, body mass index; FT, fat thickness; mm, millimeter; MT, muscle thickness; CSA, cross-sectional area; cm, centimeter.

Figure 1. ROC curves of the equation consisting of BMI, FT, MT, and CSA in predicting sarcopenia.

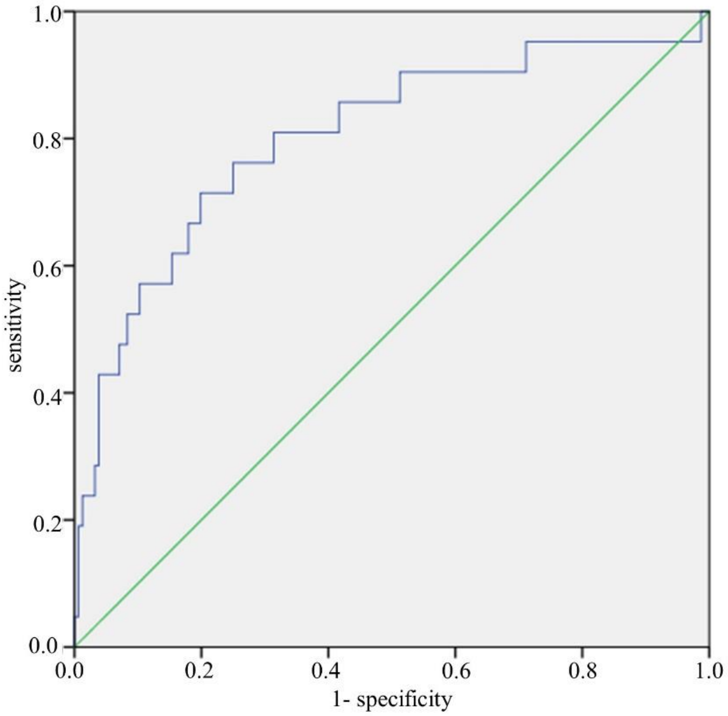

Note: AUC was 0.802 (95%CI: 0.688-0.916, P<0.001) and the prediction equation consisting of BMI, FT, MT, and CSA is: logit(P) = 2.416 – 0.095*BMI – 0.798*FT + 0.231*MT – 0.693*CSA, $P = e^{\text{logit}(P)}/1+e^{\text{logit}(P)})$. The blue line is the tracing of ROC analysis of equation in predicting sarcopenia, the green line is the baseline. Abbreviations: ROC, receiver operating characteristic.

Figure 2. ROC curves of the equation consisting of gender, age and CSA in predicting sarcopenia when grip strength of cut-off point for sarcopenia was 28 kg in male.

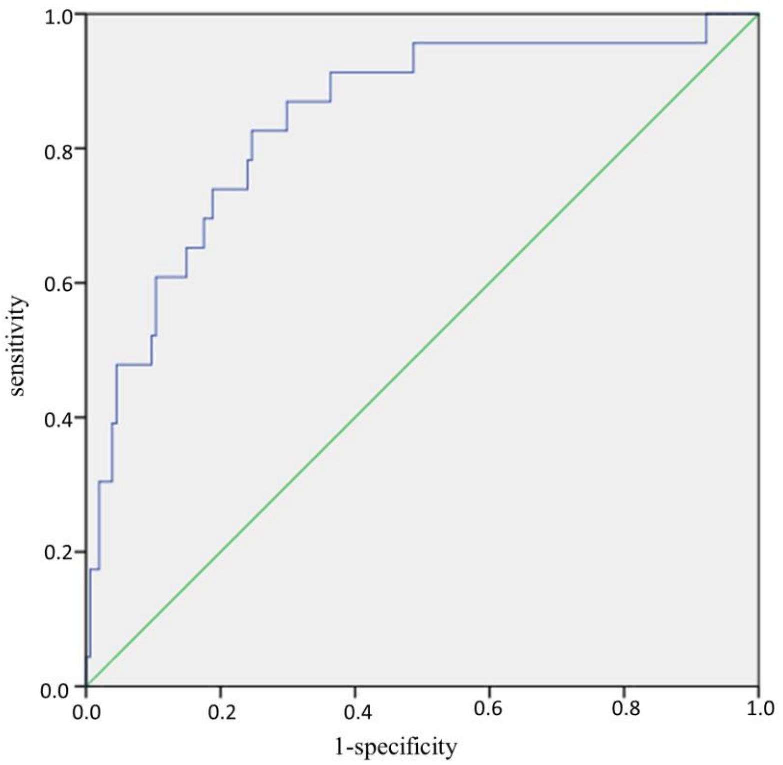

Note: when grip strength of cut-off point for sarcopenia was 28 kg in male, AUC was 0.842 (95%CI: 0.752-0.933, P<0.001) and the prediction equation is: logit(P) = –4.799 + 0.115*age – 2.585*gender (man=0, woman=1) – 0.657*CSA, $P = e^{\text{logit}(P)} / 1+e^{\text{logit}(P)}$. The blue line is the tracing of ROC analysis of equation in predicting sarcopenia, the green line is the baseline.

Abbreviations: ROC, receiver operating characteristic; AUC, area under curve.
